# A Comprehensive Review of Biologics in Phase III and IV Clinical Trials for Atopic Dermatitis

**DOI:** 10.3390/jcm13144001

**Published:** 2024-07-09

**Authors:** Katarzyna Waligóra-Dziwak, Aleksandra Dańczak-Pazdrowska, Dorota Jenerowicz

**Affiliations:** Department of Dermatology, Poznan University of Medical Sciences, 60-355 Poznań, Poland; aleksandra.danczak-pazdrowska@ump.edu.pl (A.D.-P.); dorota.jenerowicz@usk.poznan.pl (D.J.)

**Keywords:** atopic dermatitis, biological drugs, biologics, biologic therapy, clinical trials

## Abstract

Atopic dermatitis (AD) is a skin condition characterized by significant challenges and a substantial deterioration in the life quality for affected patients. The therapeutic landscape for AD has witnessed a transformative shift with the emergence of biologic therapies. Our focus centers on biologics currently undergoing phase III and IV clinical trials, deeming them to hold the highest potential for significant clinical relevance. To identify biologic drugs under development in phase III and IV clinical trials, we searched ClinicalTrials.gov. Additional relevant trials were identified through JapicCTI/ Japan Registry of Clinical Trials (jRCT) with a citation search. A search in MEDLINE and EMBASE was performed. There have been 76 clinical trials identified concerning biologic drugs: dupilumab (34 trials), lebrikizumab (14 trials), tralokinumab (10 trials), rocatinlimab (7 trials), amlitelimab (2 trials), nemolizumab (6 trials), MG-K10 (1 trial), CM310 (1 trial), 611 (1 trial). A search in MEDLINE revealed 132 articles concerning phase III and IV clinical trials for AD treatment. A total of 39 articles concerned biologic drugs covering 23 clinical trials. A search in EMBASE revealed 268 relevant articles, allowing us to identify results of an additional six clinical trials. The safety and efficacy of these biologics are comprehensively addressed in this review. This comprehensive review aims to explore the current landscape of biologic therapies for AD, delving into the latest research findings, clinical trial outcomes, and the diverse mechanisms of action employed by these novel interventions.

## 1. Introduction

Atopic dermatitis (AD) is a skin condition characterized by significant challenges and a substantial deterioration in the quality of life for affected patients. Over the years, the therapeutic landscape for AD has witnessed a transformative shift with the emergence of biologic therapies [1,2]. These innovative treatments, specifically targeting key molecular pathways involved in AD pathogenesis, have shown remarkable promise in providing substantial relief to individuals with more severe disease manifestations. In response to the unveiling of the underlying mechanisms of AD, a diverse range of treatments has emerged over the years [1,2]. 

The pathophysiology of AD is intricate, involving a combination of immune system abnormalities, genetic factors, epidermal barrier function defects, and environmental influences. The disease is driven by a T_H_2-mediated immune response, with various pathways offering potential therapeutic targets. The T_H_2 immune response is crucial in the development of AD, involving type-2 cytokines like interleukin-4 (IL-4), interleukin-5 (IL-5), interleukin-13 (IL-13), and interleukin-31 (IL-31) [2]. The diverse actions of the key cytokines IL-13 and IL-4 are facilitated by the IL-4 receptor alpha (IL-4Rα), which has become a significant therapeutic target [3,4,5]. Specifically, IL-4Rα combines with the γc chain to create a type I receptor, exclusively binding with IL-4. Additionally, IL-4Rα associates with IL-13Rα1, forming a high-affinity type II receptor that binds both IL-4 and IL-13 [4,5]. When IL-4 or IL-13 binds to these receptors, it triggers a series of events, including trans-phosphorylation and activation of Janus family protein kinases (JAKs), leading to the downstream signaling pathways associated with IL-4 and IL-13 [6]. 

Additionally, it is important to emphasize that dendritic cells expressing the OX40 ligand play a significant role in the pathophysiology of AD by binding to OX40 on T cells, thereby enhancing the production of IL-4, IL-5, IL-13, and the itch-specific cytokine IL-31. Elevated levels of T_H_2 cytokines and chemokines in the skin are consistently observed in AD patients, directly contributing to tissue inflammation, impairing the epidermal barrier, and inhibiting the production of antimicrobial peptides in response to pathogens [2].

While conventional treatments may alleviate AD symptoms temporarily, their efficacy and long-term safety are limited. As a result, sustained research efforts have explored the development of alternative drugs over the past decades, aiming to target specific pathways and cytokines such as IL-4, IL-13, or IL-31 [1,6]. In addition, other monoclonal antibodies targeting the i.a. OX40/OX40L signaling pathway are presently being investigated, with a number of them having recently commenced clinical trials [1,2]. This comprehensive review aims to explore the current landscape of biologic therapies for AD, delving into the latest research findings, clinical trial outcomes, and the diverse mechanisms of action employed by these novel interventions. Our focus centers on biologics currently undergoing phase III and IV clinical trials, deeming them to hold the highest potential for significant clinical relevance. From the first FDA-approved biologic, dupilumab, to the promising candidates undergoing clinical evaluation, this review is dedicated to elucidating the landscape of novel biological treatments for AD. 

## 2. Methods

**Figure 1:**
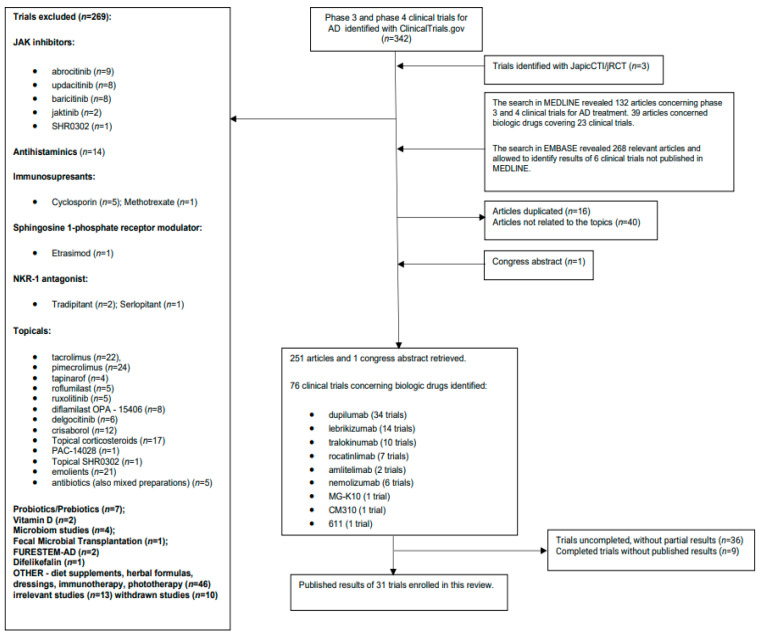
Study flowchart illustrating the search methods for the review. Abbreviations: AD, atopic dermatitis; JAK, Janus kinase; NKR-1 antagonist, neurokinin-1 receptor antagonist; *n*, number.

Biologics used in the treatment of AD were identified through Google Scholar and UptoDate. In order to identify biologic drugs under development in phase III and IV clinical trials for comprehensive review, we searched ClinicalTrials.gov (accessed on 11 January 2024) to recruit all relevant trials concerning biologics used in the treatment of AD. The search was constructed to find phase III and phase IV clinical trials for AD. We excluded trials that did not involve biologics (topical drugs, JAK inhibitors, other—Figure 1) as well as irrelevant studies (withdrawn trials, unrelated studies—Figure 1). Additional relevant clinical trials for AD were identified through JapicCTI/Japan Registry of Clinical Trials (jRCT) with a citation search. We conducted search in MEDLINE (using PubMed) and EMBASE to find publications containing phrase “atopic dermatitis”, the article type selected: “phase 3 clinical trial”/“clinical trial, phase III” or “phase 4 clinical trial”/“clinical trial, phase IV“. For additional investigation, we employed the names of biologic agents: “dupilumab”, “MG-K10”, “CM310”, “611”, “tralokinumab”, “lebrikizumab”, “nemolizumab”, “rocatinlimab”, “amlitelimab”; the article type selected: “phase 3 clinical trial”/“clinical trial, phase III” or “phase 4 clinical trial”/“clinical trial, phase IV”. Only English language studies concerning humans were enrolled in this review (Figure 1).

## 3. Results

We searched ClinicalTrials.gov in order to find phase III and phase IV clinical trials for AD with the result of 342 trials identified. We excluded trials (*n* = 269) that did not concern biologics (topical drugs, JAK inhibitors, other—Figure 1) as well as other irrelevant studies. Additional relevant clinical trials for AD were identified through JapicCTI/Japan Registry of Clinical Trials (jRCT) with a citation search (3 trials). There have been 76 clinical trials identified concerning biologic drugs: dupilumab (34 trials), lebrikizumab (14 trials), tralokinumab (10 trials), rocatinlimab (7 trials), amlitelimab (2 trials), nemolizumab (6 trials), MG-K10 (1 trial), CM310 (1 trial), 611 (1 trial) (as of 11 January 2024; Supplementary Table S1). A search in MEDLINE revealed 132 articles concerning phase III and IV clinical trials for AD treatment. A total of 39 articles concerned biologic drugs covering 23 clinical trials. A search in EMBASE revealed 268 relevant articles, allowing us to identify results of an additional six clinical trials not published in MEDLINE (as of 11 January 2024; Table 1). Additional results from two more trials have been uncovered through a congress abstract search. Identified biologic drugs were divided for further review into 4 categories, depending on the mechanism of action: (1) anti-IL-4Rα antibodies; (2) anti-IL-13 antibodies; (3) anti-IL31RA antibodies; (4) anti-OX40 antibodies.

### 3.1. Anti-IL-4Rα Antibodies

**Figure 2:**
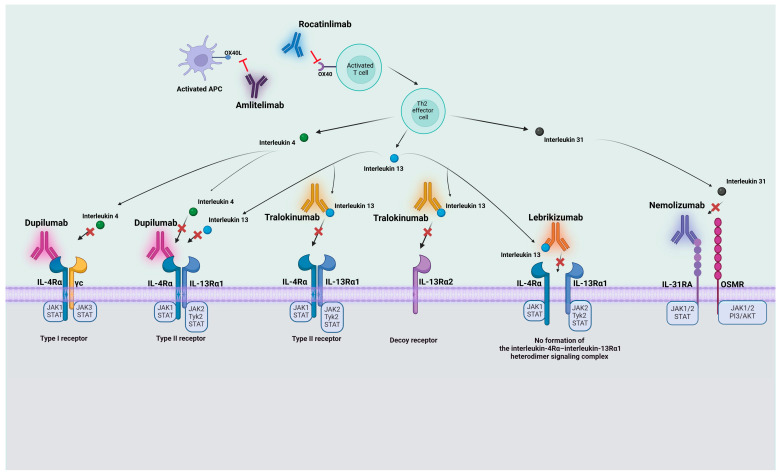
Mechanisms of action of biologic drugs for atopic dermatitis: Dupilumab, Tralokinumab, Lebrikizumab, Nemolizumab, Rocatinlimab, and Amlitelumab. Abbreviations: JAK1, Janus kinase 1; JAK2, Janus kinase 2; JAK3, Janus kinase 3; STAT, signal transducer and activator of transcription; Tyk2, Tyrosine kinase 2; IL-4Rα, IL-4 receptor alpha chain; γc, γc chain; IL-13Rα1, IL-13 receptor alpha 1 chain; IL-13Rα2, IL-13 receptor alpha 2 chain; PI3/AKT, phosphoinositide 3-kinase/protein kinase B; APC, antigen-presenting cell; IL-31RA, IL-31 receptor A; OSMR, oncostatin M receptor. Created with BioRender.com (accessed on 28 June 2024).

Biologics designed to target the IL-4 receptor alpha (IL-4Rα) have gained substantial attention due to their interaction with transmembrane protein being a critical element in the molecular mechanism of type-2 inflammation [Figure 2]. Notably, recent years have witnessed considerable progress in AD drug research and development, with a specific focus on IL-4Rα. The IL-4R complex is structured as a heterodimer, featuring a common component known as IL-4Rα. This subunit forms distinct pairings with auxiliary subunits, facilitating the diverse actions of IL-13 and IL-4 [3]. Specifically, IL-4Rα combines with the γc chain to create IL-4R type I, exclusively binding with IL-4. Additionally, IL-4Rα associates with IL-13Rα1, which is the low-affinity IL-13 binding receptor, forming a high-affinity type II heterodimeric complex, present on various cell types, that binds both IL-4 and IL-13 [4,5]. When IL-4 or IL-13 binds to these receptors, a cascade of events is initiated. This includes the trans-phosphorylation and Janus family protein kinases (JAKs) activation. Notably, JAK1 is linked with IL-4Rα chain, JAK2 is linked with IL-13Rα1 chain, and JAK3 with γc chain, contributing to the downstream signaling pathways activated by IL-4 and IL-13 [6]. Drugs designed to target IL-4Rα work by aiming to suppress signaling of both IL-4 and IL-13. Through the blockade of the IL-4Rα subunit, they effectively hinder IL-4 and IL-13 cytokine-induced responses. This inhibition extends to the release of proinflammatory cytokines and immunoglobulin E. As of now, dupilumab stands as the sole biologic therapy directed at IL-4Rα that has obtained approval from the United States Food and Drug Administration (FDA) for use in AD. However, a noteworthy current trend involves the concurrent development of drugs targeting this receptor, with several in phase III clinical trials, demonstrating favorable efficacy and safety trial data. 

#### 3.1.1. Dupilumab

Dupilumab (Dupixent^®^, Sanofi Biotechnology, Paris, France) is a fully human IgG4 monoclonal antibody that works through selective binding to IL-4Rα, effectively suppressing type 2 inflammation and relieving symptoms of AD [54,55]. Dupilumab obtained FDA approval for the treatment of moderate and severe AD in adults on March 28th, 2017, for cases when the condition is not effectively managed by topical treatments or when such therapy is not advised [56]. The dosing (adults) involves an initial administration of 600 mg subcutaneously, followed by a 300 mg dose given once every alternate week [33]. In 2022, the FDA granted approval also for the use of dupilumab in patients aged 6 months and older.

In an analysis of two phase III studies of 16 weeks duration (LIBERTY AD SOLO 1, NCT02277743; LIBERTY AD SOLO 2, NCT02277769), involving adult patients suffering from moderate and severe AD, the results were as follows: the achievement of at least 75% improvement in Eczema Area and Severity Index (EASI-75) response at week 16 was significantly higher in individuals receiving dupilumab (in monotherapy) compared to those on placebo (Table 1). Specifically, 47.7% of participants reached EASI-75, while 37% achieved an Investigator’s Global Assessment (IGA) 0 or 1 with a ≥2 point reduction from baseline at week 16, with dose administered every two weeks (Q2W). Proportions of subjects reaching EASI-50 and EASI-90 at week 16 in the dupilumab group were 67.0% and 32.8%, respectively [17]. In the LIBERTY AD CHRONOS study (NCT02260986), where concomitant corticosteroids were permitted, the EASI-75 response at week 16 was attained by 64% of subjects receiving dupilumab (plus topical corticosteroids) every week (QW). Furthermore, the EASI-75 response was observed in 69% of participants receiving dupilumab (plus topical corticosteroids) Q2W, as opposed to 23% in the control group. Comparable efficacy was sustained through week 52, with EASI-75 rates at 52 weeks being 64% and 65% in the two groups treated with dupilumab, compared to 22% in the placebo plus topical corticosteroids group [25].

Dupilumab has exhibited notable efficacy in various other studies, including those focused on children and adolescents, further substantiating its favorable therapeutic profile (Table 1). 

When safety is considered, in SOLO1 and SOLO2 trials, the most often observed adverse events (AEs) were AD exacerbations, adverse reactions at the injection site, and upper respiratory tract infections [17]. The occurrence of upper respiratory tract infections was comparable across both the dupilumab and placebo groups. Subjects treated with dupilumab experienced a slightly higher occurrence of adverse reactions at the injection site, primarily categorized as mild or moderate. In the placebo groups, AD exacerbations and various cutaneous infections were more prevalent. Incidence of allergic conjunctivitis as well as conjunctivitis with an unspecified cause was elevated in the groups treated with dupilumab compared to the groups receiving placebo [18]. Temporary rises in eosinophil counts from initial levels were observed in the dupilumab-treated groups at early weeks of the study, followed by declines to baseline by week 16. In the long-term management of AD with dupilumab (LIBERTY AD CHRONOS), in patients showing moderate to severe symptoms of the disease, no significant laboratory abnormalities attributed to dupilumab were observed. In the long term, conjunctivitis and adverse reactions at the injection site were more prevalent in dupilumab-treated patients than in subjects receiving a placebo [18,25].

#### 3.1.2. CM310

CM310 (Keymed Biosciences, Chengdu, China), a humanized IgG4 monoclonal antibody, effectively disrupts the interaction between the cytokines IL-4 and IL-13 and their co-receptor subunit IL-4Rα by specifically targeting IL-4Rα. CM310, being an experimental monoclonal antibody, has exhibited effectiveness and safety in subjects with type 2-related AD during clinical investigations. CM310 and dupilumab exhibit distinct epitopes on IL-4Rα, leading to varied cross-species reactivity. CM310 displays the capacity for interaction with IL-4Rα across human, cynomolgus monkey, and rat models, while dupilumab uniquely targets the IL-4Rα receptor in humans. This divergence suggests distinguished mechanisms in suppressing IL-4Rα signaling, potentially resulting in distinct clinical outcomes [57]. At the moment, CM310, in the form of subcutaneous injections, is undergoing evaluation in a phase III study (NCT05265923), the outcomes of which are not yet released (as of 11 January 2024). In the randomized, double-blind, placebo-controlled phase IIb study (NCT04805411), the results revealed that, at week 16, the proportions of subjects attaining EASI-75 (in the full analysis set) were 70%, 65%, and 20% in the groups receiving high-dose (HD) treatment, low-dose (LD) treatment, and placebo, respectively (in monotherapy). For EASI-50 responders at week 16, the rates were 85% in the group receiving HD treatment and 80% in the group administered LD treatment. EASI-90 responders at week 16 were 40% and 48% in the HD and LD groups, respectively. Additionally, 48% of patients achieved a ≥2-point improvement in IGA from baseline at week 16 in the HD group, while 55% of patients achieved this improvement in the LD group [58].

When safety is considered, physical assessment and vital measurements revealed no significant abnormalities. The most prevalent adverse events with an incidence of ≥10% (in both HD and LD group) included nasopharyngitis, AD, high blood lipid levels, and elevated levels of uric acid. There was no discernible imbalance in the occurrence of nasopharyngitis between the treatment and placebo groups. CM310-treated patients showed an increased frequency of hyperlipidemia and hyperuricemia. Patients administered a placebo exhibited a higher rate of AD compared to those in the treatment groups (placebo group, 10%; LD group, 8%; HD group, 0). Notably, conjunctivitis occurred at a low rate (all cases were of moderate severity) [58]. 

#### 3.1.3. MG-K10 (Comekibart) and 611

MG-K10 (Shanghai Mabgeek Biotech., Shanghai, China), also known as Comekibart, as well as an investigational drug 611 (Sunshine Guojian Pharmaceutical, Shanghai, China) are humanized monoclonal antibodies targeting IL-4Rα. Currently, both are undergoing evaluation in a multicenter, randomized, double-blind, placebo-controlled phase III trials (NCT06026891, NCT06173284). The trial findings have not yet been released (as of 11 January 2024) [59].

### 3.2. Anti-IL-13 Antibodies

IL-13, being an important mediator for T-helper type 2 inflammation, is a cytokine linked to conditions such as asthma and AD. IL-13 triggers signaling upon attachment to the IL-13 receptor alpha 1 chain (IL-13Rα1), forming a receptor complex with recruited IL-4Rα. IL-13 additionally attaches to the IL-13 receptor alpha 2 chain (IL-13Rα2), which is believed to serve an anti-inflammatory role as a decoy receptor by internalizing surplus IL-13 [60]. IL-13-neutralizing antibodies work by hindering IL-13 from attaching to its receptors. 

Tralokinumab, being a human IgG4 monoclonal antibody, achieves IL-13 neutralization by specifically targeting helices A and D of IL-13, thereby preventing IL-13 from interacting with IL-13Rα1 and IL-13Rα2. The studies confirm that tralokinumab effectively hinders the biological actions of IL-13 through this specific binding mechanism [61].

Apart from tralokinumab, another antibody directed at IL-13, undergoing evaluation in late stages of clinical trials, is lebrikizumab. These two biological treatments focus on distinct epitopes on IL-13, causing interference with IL-13 communication pathways through unique approaches. Through its interaction with IL-13, lebrikizumab disrupts the creation of the IL-4Rα–IL-13Rα1 heterodimeric complex, in that way blocking IL-13 signaling [42,62]. While both antibodies effectively block signaling through IL-13Rα1, tralokinumab hinders the attachment of IL-13 to IL-13Rα2, a feature not shared by lebrikizumab. Not preventing the interaction of IL-13 with the IL-13Rα2, which is considered a decoy receptor, allows for internalizing of excess IL-13 in case of lebrikizumab treatment. Notably, while tralokinumab as well as lebrikizumab employ unique approaches to counter IL-13, they both have demonstrated efficacy in managing AD [42].

#### 3.2.1. Tralokinumab

Tralokinumab is a monoclonal antibody directed at IL-13. It is categorized as a fully human IgG4 antibody, which targets specifically IL-13, neutralizing it and as a result inhibiting type 2 inflammation [63]. Recent findings have revealed that IL-13, characterized as a type 2 cytokine, constitutes a critical factor responsible for the underlying inflammation in AD [64] (Figure 2). In 2021, the FDA granted approval for tralokinumab (Adbry^®^/Adtralza^®^, LEO Pharma, Ballerup, Denmark), which was also approved by the European Commission in the same year for the management of moderate and severe AD in patients aged ≥12 years, for whom topical treatments are inadequate. The prescribed regimen consists of an initial dose of 600 mg, administered as four subcutaneous injections of 150 mg each, followed by 300 mg (administered as two subcutaneous injections) Q2W [65]. The assessment of effectiveness is advised after a 16-week period, and discontinuation should be considered if there is no observable response. Patients achieving satisfactory improvement after 16 weeks of treatment may consider switching to a dosing interval of Q4W, although this adjustment may not be suitable for individuals weighing over 100kg. Additionally, tralokinumab is permissible for use in combination with a topical steroid [40]. 

When efficacy is considered, in studies where tralokinumab was investigated as monotherapy, specifically, ECZTRA 1 and ECZTRA 2 (NCT03160885; NCT03131648), an IGA score of 0 or 1 was attained after 16 weeks by 15.8% of tralokinumab-treated patients compared to 7.1% of placebo-receiving patients in ECZTRA 1, and by 22.2% of tralokinumab-treated patients versus 10.9% of placebo-receiving patients in ECZTRA 2 [36].

In ECZTRA 1, 25.0% of tralokinumab-treated patients attained EASI-75 after 16 weeks of treatment in comparison to 12.7% of placebo-receiving patients. In ECZTRA 2, after the same treatment period, 33.2% of tralokinumab-treated patients achieved EASI-75 versus 11.4% of placebo-receiving individuals. EASI-90 was achieved by 14.5% of tralokinumab-treated patients versus 4.1% of placebo-receiving patients in ECZTRA 1 and by 18.3% of tralokinumab-treated patients versus 5.5% of placebo-receiving patients in ECZTRA 2. EASI-50 at week 16 was achieved by 41.6% and 49.9% with tralokinumab (vs. 21.3% and 20.4% with placebo) in ECZTRA 1 and ECZTRA 2, respectively [36]. 

When considering long-term outcomes, in ECZTRA 1 and ECZTRA 2 studies (tralokinumab in monotherapy), a group of 185 and 227 patients were rerandomized in a 2:2:1 ratio to participate in a 52-week study. They were either assigned to continue tralokinumab Q2W, or to change the tralokinumab dosing interval to Q4W, or to transition to a placebo regimen. Among patients who attained an IGA 0/1 with tralokinumab by week 16, the maintenance of an IGA 0/1 at week 52 was noted in 51% with an unchanged treatment regimen (continued tralokinumab Q2W) compared to 47% with transition to a placebo regimen in ECZTRA 1. In ECZTRA 2, the corresponding percentages were 59% with an unchanged treatment regimen and 25% with transition to a placebo regimen [36]. EASI-75 was maintained by 60% of patients with an unchanged treatment regimen versus 33% of patients with transition to a placebo regimen in ECZTRA 1 and the corresponding percentages in ECZTRA 2 were 56% with continued treatment versus 21% with placebo transition [36]. The efficacy of tralokinumab has been demonstrated in several other studies, including those focused on adolescents (Table 1).

Based on pooled analysis of five randomized, double-blind, placebo-controlled trials (NCT02347176, NCT03562377, NCT03131648, NCT03160885, NCT03363854) [37], favorable tolerability of tralokinumab was demonstrated. The vast majority of AEs fell within the categories of mild or moderate severity, and a significant portion of them had resolved completely by the conclusion of the treatment duration. Only a limited number of patients encountered AEs severe enough to necessitate the permanent cessation of treatment.

Throughout the initial 16-week treatment phase, the occurrence rates of any AEs, including serious AEs, were comparable for tralokinumab-treated and placebo-receiving patients. The incidence of AEs did not escalate with continued treatment over the course of 52 weeks. Common AEs included nasopharyngitis, conjunctivitis, and adverse reactions at the injection site (observed with greater frequency in the tralokinumab group). Notably, conjunctivitis as an AE of special interest was observed more often in tralokinumab-treated patients. The majority of cases of conjunctivitis were of mild severity and resolved by the conclusion of the trial duration, although a single case led to therapy discontinuation. Certain events occurred at a lower rate in the tralokinumab-treated group compared to placebo, namely cutaneous infections necessitating systemic treatment, eczema herpeticum, and opportunistic and serious infections. Physical assessments, vital sign measurements, and electrocardiograms showed no notable abnormalities. Additionally, there were no significant laboratory abnormalities observed. Even though the tralokinumab group patients suffered eosinophilia at a higher rate during the initial weeks of the study, in the long-term monitoring, mean eosinophil levels returned to baseline. The safety characteristics of patients with eosinophilia were in line with those of the entire trial population [37]. 

#### 3.2.2. Lebrikizumab

Lebrikizumab is an anti-IL-13 humanized monoclonal antibody. It specifically binds to IL-13, disrupting the formation of the IL-4Rα–IL-13Rα1 heterodimer signaling complex [66]. In November 2023, lebrikizumab (Ebglyss^®^, Alimrall, Barcelona, Spain) received approval from the European Commission for the management of moderate to severe AD in patients aged ≥12 years who meet the requirements for systemic treatment and weigh more than 40 kg. Lebrikizumab has not yet received approval from the FDA (as of January 2024). The recommended dosage regimen for lebrikizumab involves a starting dose of 500 mg (administered as two subcutaneous injections of 250 mg each) at both week 0 and week 2. Following this, a subcutaneous injection of 250 mg Q2W is administered until week 16. Consideration of treatment discontinuation is advised for patients demonstrating a lack of satisfactory response by week 16. For those with an initial partial response, ongoing treatment Q2W up to week 24 may lead to further improvement. Upon achieving a clinical response, the maintenance dose is 250 mg administered Q4W. 

When evaluating efficacy, clinical trials ADvocate1 (NCT04146363) and ADvocate2 (NCT04178967), where lebrikizumab was examined as monotherapy, yielded promising results. At week 16, in ADvocate1, 43.1% of lebrikizumab-treated patients achieved IGA 0/1 (≥2-point reduction), in contrast to 12.7% in the placebo group. In ADvocate2, the corresponding percentages were 33.2% for the lebrikizumab-treated patients and 10.8% for those receiving placebo [42]. In both trials of ADvocate1 and ADvocate2, a higher proportion of lebrikizumab-treated subjects attained an EASI-75 response by week 16 compared to the placebo-receiving patients. Specifically, in ADvocate1, 58.8% of lebrikizumab-treated subjects achieved an EASI-75 response, contrasting with 16.2% in the placebo-receiving group. Similarly, in ADvocate2, the corresponding percentages were 52.1% for the lebrikizumab-treated patients and 18.1% for the placebo-receiving individuals. Furthermore, in ADvocate1, 38.3% of lebrikizumab-treated patients achieved an EASI-90 response versus 9.0% of placebo-receiving patients (week 16 results). In ADvocate2, the corresponding percentages were 30.7% and 9.5%, respectively [43]. At week 52, a sustained IGA 0/1 was observed in 71.2% of subjects undergoing treatment Q2W, 76.9% of those receiving treatment Q4W, and 47.9% of patients with transition to a placebo regimen. Additionally, the maintenance of EASI-75 response rates at 52 weeks was demonstrated in 78.4% of lebrikizumab-treated patients Q2W, 81.7% of patients receiving treatment Q4W, and 66.4% of patients with transition to a placebo regimen [43].

The efficacy of lebrikizumab has been demonstrated in several other studies, including those focused on adolescents as well as evaluating immune responses to vaccines (Table 1). 

An integrated analysis of eight clinical trials (NCT02465606, NCT04392154, NCT02340234, NCT04178967, NCT04250337, NCT04250350, NCT04146363, NCT03443024) [67] indicated favorable tolerability of lebrikizumab. Most cases of AEs were mild or moderate in severity, and usually did not necessitate treatment discontinuation. Notably, conjunctivitis emerged as the common AE and was more frequently observed in lebrikizumab-treated patients during the placebo-controlled period. Most of conjunctivitis cases were unserious, with only a few requiring treatment cessation. The occurrence of conjunctivitis (cluster) was 8.5% in lebrikizumab-treated Q2W groups (LEBQ2W) versus 2.5% in the group receiving placebo. Infections occurred at a similar rate (18.9% in placebo group and 21.2% in LEBQ2W groups) and were mostly nonserious in both the placebo and lebrikizumab groups, with only a few resulting in study intervention discontinuation. No opportunistic infections were observed. Lebrikizumab-treated patients showed a lower rate of cutaneous infections compared to the placebo group (2.2% vs. 5.9%). Herpes zoster cases were uncommon in the lebrikizumab-treated patients, with a lower occurrence compared to patients treated with JAK inhibitors. However, they occurred with a slightly higher frequency than in the placebo-treated individuals (0.6% in LEBQ2W group vs. 0% in the placebo group). When laboratory results are considered, mean blood eosinophil count was reported to increase slightly from baseline in lebrikizumab group [67]. 

### 3.3. Anti-IL31RA Antibodies

Research conducted in animal models suggests that IL-31 may play a critical part in the pruritus pathomechanism [68]. Elevated expression of IL-31 has been demonstrated to be linked with the stimulation of sensory neuronal outgrowth [69], enhancing sensitivity to even minor stimuli that induce itching, leading to persistent pruritus. IL-31 skin expression is further connected to a substantial repression of the filaggrin protein. Filaggrin plays a vital part in the differentiation of keratinocytes and the preservation of the skin barrier [70]. The receptor for IL-31 is a heterodimeric complex consisting of IL-31 receptor A (IL-31RA) and the oncostatin M receptor (OSMR) [71]; when IL-31 binds to its receptor, it initiates cellular signaling pathways, including the Janus kinase/signal transducer and activator of transcription (Jak/STAT), phosphoinositide 3-kinase/protein kinase B (PI3K/AKT), and mitogen-activated protein kinase (MAPK) [71]. IL-31 has been demonstrated to prompt cell cycle arrest within keratinocytes, causing a decrease in proliferation. This contributes to abnormal skin development as well as impaired barrier function [72]. The recent data have indicated the crucial role of IL-31 in the clinical presentation of acute and persistent itching [73,74].

#### Nemolizumab

Nemolizumab is a subcutaneously administered humanized anti-IL-31RA IgG2k monoclonal antibody [75]. In March 2022, Nemolizumab (Mitchga^®^ Syringes, Maruho, Osaka, Japan), received approval in Japan for treating itch linked to AD in adult patients and children aged ≥13 years. This approval is granted when existing treatments prove to be insufficiently effective [75]. In December 2019, the FDA bestowed Breakthrough Therapy designation upon Nemolizumab for addressing itch related to prurigo nodularis. As of January 2024, Nemolizumab awaits the FDA’s approval. Nemolizumab in Japan is approved for subcutaneous use in a dose of 60 mg every 4 weeks [76,77]. 

During the ARCADIA 1 and ARCADIA 2 studies (NCT03985943, NCT03989349) [53], participants received subcutaneously a loading dose of 60 mg nemolizumab at the baseline. Subsequently, they received a 30 mg nemolizumab subcutaneous injection every four weeks. Those identified as responders underwent randomization into three arms (1:1:1): either continuing with 30 mg nemolizumab Q4W or 30 mg nemolizumab once every eight weeks (Q8W), or transitioning to an injection of placebo. Findings from the ARCADIA 1 and 2 trials showcased the effectiveness of nemolizumab in management of AD symptoms and alleviating pruritus among adolescent and adult patients with moderate or severe AD. The studies were placebo-controlled, with both groups receiving concurrent therapy with topical calcineurin inhibitors or topical corticosteroids. Across both studies, individuals treated with nemolizumab exhibited statistically significant enhancements in key outcome measures relative to those receiving a placebo, following a 16-week treatment period [53]. IGA 0/1 achieved 35.6% and 37.7% of subjects in nemolizumab groups in ARCADIA 1 and 2, respectively, in comparison to 24.6% and 26.0% in the groups receiving placebo. Furthermore, 43.5% and 42.1% of patients in nemolizumab groups in ARCADIA 1 and 2, respectively, attained an EASI-75, in comparison to 29.0% and 30.2% in the placebo-receiving groups [53]. Both studies successfully addressed secondary outcome measures. Specifically, in ARCADIA 1 and 2, 48.6% and 48.1% of patients, respectively, treated with nemolizumab achieved itch reduction of at least four points, quantified by the peak-pruritus numerical rating scale (PP-NRS) score. This surpassed the corresponding rates of 20.5% and 20.6% observed in the control group within a 16-week therapy duration. Furthermore, statistically significant results were evident not only at week 16 but also earlier, underscoring the rapid onset of nemolizumab’s efficacy in addressing both itch and sleep disturbances [53]. In another phase III trial (JapicCTI number, 173740), examining the use of subcutaneous nemolizumab with concurrent use of topical glucocorticoids and moisturizing agents [78], at week 16, the least-squares mean percent change from baseline in the pruritus visual analogue scale (VAS) score was −42.8% in the group treated with nemolizumab, compared to −21.4% in the group receiving a placebo. When considering the EASI score, the least-squares mean percent change at week 16 was −45.9% in the nemolizumab-treated group, compared to −33.2% in the control group [78] (Table 1).

When safety is considered, on the basis of meta-analysis [50] (which comprised 569 subjects who were administered nemolizumab and 240 patients receiving placebo), considering six studies analyzed [69,79,80,81,82,83], it might be concluded that nemolizumab is characterized by generally good tolerability. Among patients treated with nemolizumab, the most frequently reported AEs included infections and skin or subcutaneous tissue disorders, nasopharyngitis (reported in 10–32.7% of subjects) as well as AD exacerbations (reported in 15–28.1% of subjects). There were also noted occurrences of respiratory and gastrointestinal adverse events, thoracic and mediastinal disorders as well as adverse reactions at the injection site in both nemolizumab and placebo-treated patients. Importantly, the incidences of these events were relatively small and exhibited comparable percentiles between the two groups. Furthermore, the severity of AEs was generally nonserious, with only a few being categorized as severe [50].

### 3.4. Anti-OX40 Antibodies

In the pathogenesis of AD, the interaction between OX40 and OX40L, representing two co-stimulatory immune checkpoint molecules, assumes an important role. Immune checkpoint molecules have co-stimulatory and co-inhibitory functions within adaptive immune responses. These molecules can be broadly categorized into the following superfamilies: (1) the immunoglobulin superfamily and (2) the tumor necrosis factor superfamily and its receptors. Notably, OX40 and its ligand OX40L emerge as the co-stimulatory immune checkpoint molecules being part of the second superfamily [84,85]. The co-stimulatory T-cell receptor OX40 exhibits predominant expression on effector and regulatory T-cells [85]. Its corresponding ligand, OX40L, is found on various activated antigen-presenting cells, including dendritic cells, macrophages, activated B-cells, and endothelial cells. The interaction between OX40 and OX40L is pivotal for enhancing the expansion of effector T-cells and prolonging their survival by apoptosis suppression. This engagement also plays an important role in amplifying T-cell effector activities, such as cytokine secretion, and contributes to the generation of T helper memory cells [85]. Naïve T-cells undergo activation upon interaction with antigen-presenting cells through co-stimulatory molecules, such as CD80/CD86 and CD28. The sustained expansion of activated effector Th1 and Th2 T-cells is facilitated by the ligation of OX40–OX40L [85]. Although resting memory T-cells do not express OX40, they transform into effector memory T-cells upon reactivation, subsequently expressing OX40. This OX40–OX40L ligation significantly enhances the expansion of these cells [84,85,86]. Early-stage investigations utilizing models of asthma and dermatitis have provided evidence supporting the vital role of OX40–OX40L signaling interactions in governing the effectiveness of responses regulated by memory Th2 cells [87,88]. Dendritic cells expressing OX40L play a crucial part in inducing the differentiation of OX40-positive T-cells, including Th2 cells, which stimulate the production of IL-4 and IL-13 from T-cells [89,90].

Among those afflicted with AD, the surface presentation of OX40 and OX40L on mononuclear cells in peripheral blood was elevated when contrasted with the levels observed in healthy subjects. Significant associations were noted between the scores reflecting AD activity and markers linked to the Th2 response and OX40L [85,86].

#### 3.4.1. Rocatinlimab

Rocatinlimab (Kyowa Kirin, Tokyo, Japan), initially identified as AMG 451/KHK4083, stands as a fully human IgG1 anti-OX40 monoclonal antibody presently undergoing assessment for the moderate and severe AD management within the context of phase III clinical trials. Demonstrating its efficacy, it has proven its ability for selective depletion of OX40+ activated T-cells and suppression of clonal T-cells [85,91]. Anticipated to exert control over Th2-mediated disorders, rocatainlimab represents a promising candidate in the pursuit of advanced therapeutic options for AD [85,91] (Figure 2). 

At present, seven phase III studies are in progress to assess the effectiveness and safety profile of rocatinlimab as well as to assess various treatment schemes (Supplementary Table S1), the results of which are not yet available. 

A phase IIb multicenter, randomized clinical trial (NCT03703102) was designed to evaluate the effectiveness and safety profile of rocatinlimab in individuals suffering from AD, with baseline EASI ≥ 16 and an insufficient response to topical interventions [92,93]. The 274 subjects underwent randomization into five arms: rocatinlimab 300 mg Q2W, 600 mg Q2W, 150 mg Q4W, 600 mg Q4W, or a placebo (subcutaneous). The Rocatinlimab cohorts underwent a 36-week treatment regimen, succeeded by a 20-week period of post-treatment observation, whereas the placebo cohort initially were administered a placebo for eighteen weeks. Subsequently, individuals receiving a placebo transitioned to an 18-week course of rocatinlimab treatment (600 mg Q2W), followed by a 20-week off-drug monitoring phase. By week 16, every rocatinlimab group demonstrated a meaningful improvement in the EASI score (reduction ranging from −48.3% to −61.1%) compared to the placebo (−15.0%). Within the rocatinlimab-treated group, 36.5% to 55.8% exhibited a 4-point improvement or more from baseline in the pruritus Numerical Rating Scale (NRS) score, surpassing the placebo group (19.3%). Rocatinlimab groups demonstrated increased percentages of patients achieving EASI-75 (44.2%, 40.4%, 53.8%, and 38.9%, respectively), in contrast to the placebo group (10.5%). Noteworthy improvements were particularly evident after 16 weeks of treatment for the 300 mg Q2W dosage of rocatinlimab in comparison to other therapeutic schemes. The effectiveness patterns persisted beyond week 16 for all rocatinlimab cohorts, reaching peak responses for the group receiving 300 mg Q2W (EASI-75 achieved 65.4% and 63.5% at weeks 24 and 36, respectively) [85]. In terms of safety assessment up to week 18, AEs were observed in 81% of rocatainlimab-treated individuals compared to 72% of placebo-receiving subjects. Notably, the most frequently reported AEs in rocatainlimab-treated subjects included pyrexia (17% of patients), nasopharyngitis (14% of patients), chills (11% of patients), headache (9% of patients), aphthous ulcer (7% of patients), and nausea (6% of patients) [92].

#### 3.4.2. Amlitelimab

Amlitelimab (Sanofi) is an IgG4 fully human monoclonal antibody that acts by binding to OX40L on antigen-presenting cells [85]. As a result, OX40–OX40L signaling is disrupted (Figure 2). 

At present, two phase III studies (NCT06130566, NCT06181435) are in progress to assess the effectiveness and safety profile of amlitelimab (Supplementary Table S1), the results of which are not yet available. 

In a phase IIb trial, AD patients received amlitelimab subcutaneously in various dosing regimens (62.5 mg, 125 mg, 250 mg Q4W or 250 mg Q4W with loading dose). Improvements in percentage change in EASI from baseline to week 16 vs. placebo were observed (difference from placebo in least squares mean change from baseline: 30.2% for 62.5 mg group; 22.2% for 125 mg group; 27.3% for 250 mg group; 32.1% for 250 mg with loading dose group) [94]. 

All treatment regimens were well tolerated, although detailed information on AE is not yet fully available [94].

Overall, the findings from clinical trials underscore the potential of targeting the OX40–OX40L signaling pathway as an innovative strategy for treating AD.

## 4. Discussion

As biologic drugs continue to play an increasingly pivotal role in clinical practice, informed decision making based on clinical trial data is imperative. Our comprehensive review aimed to facilitate this process by meticulously gathering data from diverse sources, including ClinicalTrials.gov, JapicCTI/jRCT (with citation search), MEDLINE, and EMBASE. We aimed to present this data in a concise tabular format, detailing treatment regimens and study results at specific time points. This approach is designed to provide clinicians with clear insights into the efficacy and safety profiles of various biologics.

However, it is important to note that we were not able to include results for certain novel biologics in late stages of development, such as amlitelimab or rocatinlimab, where clinical trial outcomes are still pending. Furthermore, our focus on biologics meant that we did not encompass studies involving other promising interventions, such as he-ad-to-head comparisons with non-biologic treatments like JAK inhibitors.

Further research could investigate these areas to expand our knowledge of treatment options in dermatological practice. While our review concentrated on biologics and their clinical trial outcomes, there remains a need for continued exploration and evaluation of emerging therapies to optimize patient care in dermatology.

## 5. Conclusions and Future Perspective

The landscape of biologic drugs in treating AD has witnessed a remarkable transformation, marking the onset of a new era characterized by targeted therapies. The quest for innovative therapeutics to treat AD represents one of the most dynamic areas in dermatological research, encompassing not only biologics but also small molecules and other emerging approaches. Currently, the most extensively studied biological targets include IL-4Rα, IL-13, IL31RA, and OX40–OX40L. Nevertheless, we are witnessing the rapid emergence of new therapeutic targets. At the moment, dupilumab, alongside newly developed monoclonal antibodies like tralokinumab, lebrikizumab, nemolizumab, CM-310, MG-K10, 611, amlitelimab, and the promising rocatinlimab, currently advancing through late clinical stages, create a constellation of biologics, each distinguished by its unique mechanisms of action, demonstrating notable efficacy in navigating the intricate pathophysiology of AD. As we await the results of ongoing trials and anticipate the potential approval of these next-generation biologics, it is evident that they hold considerable promise in reshaping the treatment paradigm for AD, offering hope for improved symptom management and a better quality of life for those affected by this chronic and often challenging condition. The ongoing commitment to research and innovation in the realm of biologic therapies underscores a collective dedication to advancing the care and outcomes for individuals suffering from AD. 

## Figures and Tables

**Table 1:** Summary of published (MEDLINE, EMBASE, congress abstract search, as of 11 January 2024) phase III and IV clinical trials on biologic drugs in AD treatment. Phase III and IV studies without published results are not included.

Study No.	Clinical Trial Phase	No. of Patients	Treatment	Results	Ref. No.
Anti-IL-4Rα antibodies					
			DUPILUMAB		
NCT04033367 (DUPISTAD)	Phase IV	188; Adults	Dupilumab 300 mg Q2W or placebo Q2W for 12 weeks; Patients then entered an open-label phase and received dupilumab 300 mg Q2W for 12 weeks. TCS permitted	Week 12 results: Dupilumab demonstrated a substantial enhancement in sleep quality as opposed to a placebo (with a LSMD of −15.5%), and no unfavorable alterations were observed in routine laboratory parameters.	[7]
NCT03345914 (LIBERTY AD PEDS)	Phase III	367; Children aged 6–11	Dupilumab 300 mg (Q4W) + TCS, or a weight-based regimen of dupilumab 100 mg (baseline weight < 30 kg) or 200 mg (baseline weight ≥ 30 kg) Q2W + TCS, or placebo + TCS	Week 16 results: IGA 0-1: 300 mg Q4W + TCS (32.8%), 100/200 mg Q2W + TCS (29.5%) vs. placebo + TCS (11.4%) EASI-75: 300 mg Q4W + TCS (69.7%), 100/200 mg Q2W + TCS (67.2%), placebo + TCS (26.8%) Posology was proposed for children (300 mg Q4W when weight 15–< 30 kg, 200 mg Q2W when weight 30–< 60 kg)	[8] [9] [10] [11]
NCT03346434 (Liberty AD PRESCHOOL)	Phase III	162; Children aged 6 months to <6 years	Dupilumab (5 kg to <15 kg: 200 mg; 15 kg to <30 kg: 300 mg) Q4W + TCS or placebo + TCS	Week 16 results: IGA 0-1: Dupilumab group 28% vs. Placebo group 4% EASI-75: Dupilumab group 53% vs. Placebo group 11% No clinically meaningful changes in laboratory parameters were observed.	[12,13]
NCT02612454 (LIBERTY AD PED-OLE)	Phase III	294; Adolescents aged ≥12 to <18 years	A weight-based regimen of dupilumab (2 or 4 mg/kg every week). Following protocol amendment: dupilumab 300 mg Q4W. Patients with an inadequate clinical response: dose regimens of 200 or 300 mg Q2W. Concomitant TCS permitted.	Week 52 results: IGA 0-1: Dupilumab group 42.7% EASI-75: Dupilumab group 81.2%	[14] [15]
NCT03912259	Phase III	165; Adults	Dupilumab 300 mg or placebo Q2W for 16 weeks. (monotherapy)	Week 16 results: IGA 0-1: Dupilumab group (26.8%) vs. placebo group (4.8%) EASI-75: Dupilumab group (57.3%) vs. placebo group (14.5%)	[16]
NCT02277743 (SOLO1) NCT02277769 (SOLO2) pooled data	Phase III	1379; Adults	Dupilumab 300 mg Q2W or Dupilumab 300 mg QW or Placebo QW (monotherapy)	Week 16 results: IGA 0-1 (≥2-point reduction): Dupilumab 300 mg Q2W (37.0%), Dupilumab 300 mg QW (36.8%), Placebo (9.3%) EASI-75: Dupilumab 300 mg Q2W (47.7%), Dupilumab 300 mg QW (50.2%), Placebo (13.3%) Patients with IGA > 1 at week 16 experienced significant EASI improvement with dupilumab compared to placebo (−48.9% vs. −11.3%). At the treatment period’s end, LS mean percent changes in SCORAD were −54.3 with dupilumab vs. −24.0 with placebo. Dupilumab positively impacted sleep, pain, anxiety, itch, and depression. Notably, significant clinical improvement and favorable tolerability were observed across White, Asian, and Black/African American racial subgroups.	[17] [18] [19] [20] [21] [22] [23]
NCT02755649 (CAFÉ)	Phase III	325; Adults with an inadequate response or intolerance to ciclosporin A or when this treatment is medically inadvisable	Dupilumab 300 mg Q2W + TCS or Dupilumab 300 mg QW + TCS or Placebo QW + TCS	Week 16 results: IGA 0-1 (≥2-point reduction): Dupilumab Q2W + TCS (40.2%), Dupilumab QW + TCS (39.1%), Placebo + TCS (13.9%) EASI-75: Dupilumab Q2W + TCS (62.6%), Dupilumab QW + TCS (59.1%), Placebo + TCS (29.6%) At the end of the treatment period, the LS mean percent changes in SCORAD were −58.3 with dupilumab vs. −29.5 with placebo.	[19] [24]
NCT02260986 (CHRONOS)	Phase III	740; Adults	Dupilumab 300 mg Q2W + TCS or Dupilumab 300 mg QW + TCS or Placebo QW + TCS	Week 16 results: IGA 0-1: Dupilumab QW + TCS (39%), Dupilumab Q2W + TCS (39%), Placebo (12%) EASI-75: Dupilumab QW + TCS (64%), Dupilumab Q2W + TCS (69%), Placebo (23%) Week 52 results: IGA 0-1: Dupilumab QW + TCS (40%), Dupilumab Q2W + TCS (36%), Placebo (12.5%) EASI-75 at 52 weeks: Dupilumab groups (64%, 65%), Placebo group (22%) At the end of the treatment period, the LS mean percent changes in SCORAD were −69.4% with dupilumab vs. −47.3% with placebo. Positive effect on itch was observed. Notably, significant clinical improvement and favorable tolerability were observed across White, Asian, and Black/African American racial subgroups.	[25] [19] [26] [21] [22]
NCT03054428 (LIBERTY AD ADOL)	Phase III	251; Adolescents aged ≥12 to <18 years	Dupilumab 300 mg Q4W, or Dupilumab 200 or 300 mg Q2W (<60 kg or ≥60 kg, respectively) or placebo; (monotherapy)	Week 16 results: IGA 0-1: Q2W (24.4%), Q4W (17.9%) vs. placebo (2.4%) EASI-75: Dupilumab 300 mg Q4W (38.1%), Dupilumab 200/300 mg Q2W (41.5%) vs. placebo (8.2%) When prior immunosuppressant therapy considered: EASI-75 at week 16: 33.3% with prior use of systemic immunosuppressant (SIS); 51.4% without prior SIS. Posology was proposed for adolescents: (200/300 mg Q2W; when weight 30–<60 kg/≥60 kg) No clinically meaningful changes in laboratory parameters were seen in adolescents. Positive effect on itch observed.	[9] [27] [28] [21] [29] [30]
NCT01949311 (LIBERTY AD OLE)	Phase III	Of 2677 patients enrolled, 347 reached week 148; Adults	Dupilumab 200 mg QW. Following protocol amendment: Dupilumab 300 mg QW TCS permitted	Week 148 results: IGA 0-1: 74.1% of patients EASI-75: 96.6% of patients	[31] [32]
NCT02395133 (LIBERTY AD SOLO-CONTINUE)	Phase III	422; Adults	Responding patients treated with dupilumab in SOLO were rerandomized 2:1:1:1 to: - original regimen of dupilumab, - 300 mg QW or Q2W - 300 mg, Q4W or Q8W - placebo for 36 weeks; (monotherapy)	More patients taking dupilumab QW or Q2W (71.6%) maintained EASI-75 response than those taking dupilumab Q4W (58.3%) or Q8W (54.9%) or those taking placebo (30.4%)	[33]
NCT04417894 (LIBERTY-AD-HAFT)	Phase III	133; Adults and adolescents (≥12 years)	Dupilumab 300 mg Q2W in adults; 200/300 mg Q2W in adolescents, or placebo (monotherapy)	Week 16 results: IGA 0-1: 40.3% dupilumab vs. 16.7% placebo HECSI-75: 46.9% dupilumab vs. 21.5% placebo	[34]
NCT03687359 (PEDISTAD)	Phase III	214 (dupilumab); Children aged 6 months to 11 years	Dose at discretion of study investigator (real-world registry)	3 years results: The mean (± SE) EASI score decreased with dupilumab from 19.7 ± 1.0 at start to 6.1 ± 0.8 at last observation	[35]
**Anti-IL-13 antibodies**					
			TRALOKINUMAB		
NCT03131648 (ECZTRA 1)	Phase III	802; Adults	Tralokinumab 300 mg (Q2W) or placebo (monotherapy) After 16 weeks, 185 patients were rerandomized 2:2:1 to: - continue tralokinumab Q2W, - tralokinumab Q4W, - placebo Q2W	Week 16 results: IGA 0-1: 15.8% tralokinumab vs. 7.1% placebo EASI-75: 25.0% tralokinumab vs. 12.7% placebo Week 52 results: In patients who achieved IGA 0 or 1 with tralokinumab at week 16, IGA 0-1: maintained by 51% patients with continued tralokinumab Q2W vs. 47% with tralokinumab Q2W to placebo EASI-75: maintained by 60% patients with continued tralokinumab Q2W vs. 33% with tralokinumab Q2W to placebo	[36]
NCT03160885 (ECZTRA 2)	Phase III	794; Adults	Tralokinumab 300 mg (Q2W) (monotherapy) After 16 weeks, 227 patients were rerandomized 2:2:1 to: - continue tralokinumab Q2W, - tralokinumab Q4W, - placebo Q2W	Week 16 results: IGA 0-1: 22.2% tralokinumab vs. 10.9% placebo EASI-75: 33.2% tralokinumab vs. 11.4% placebo Week 52 results: In patients who achieved IGA 0 or 1 with tralokinumab at week 16, IGA 0-1: maintained by 59% patients with continued tralokinumab Q2W vs. 25% with tralokinumab Q2W to placebo EASI-75: maintained by 56% patients with continued tralokinumab Q2W vs. 21% with tralokinumab Q2W to placebo	[36]
NCT03363854 (ECZTRA 3)	Phase III	380; Adults	Tralokinumab 300 mg or placebo Q2W for 16 weeks. At Week 16, patients who achieved IGA 0-1 and/or EASI-75 were re-randomized 1:1 to tralokinumab Q2W or Q4W. TCS allowed as needed. Patients not achieving the clinical response criteria: tralokinumab Q2W + TCS from week 16	Week 32 results: IGA 0-1: Tralokinumab Q2W/Q4W + TCS (48.8%) EASI-75: Q2W/Q4W + TCS (70.2%) Good tolerability and safety.	[37] [38]
NCT03526861 (ECZTRA 6)	Phase III	289; Adolescentsaged 12 to 17 years	Tralokinumab, 150 or 300 mg, or placebo Q2W (monotherapy)	Week 16 results: IGA 0-1: Tralokinumab 150 mg (21.4%), 300 mg (17.5%), Placebo (4.3%) EASI-75: Tralokinumab 150 mg (28.6%), 300 mg (27.8%), Placebo (6.4%)	[39]
NCT03761537 (ECZTRA 7)	Phase III	277; Adults with inadequate response to or intolerance of ciclosporin A	Tralokinumab 300 mg or placebo Q2W + TCS as needed	Week 16 results: EASI-75: Tralokinumab (64.2%) vs. Placebo (50.5%)	[40]
NCT03587805 (ECZTEND)	Phase III	345; Adults Inclusion of participants regardless of prior level of response. Nevertheless, participants with a good response might be more likely to enroll.	Tralokinumab 300 mg + TCS/TCI, Q2W	2 years results: IGA 0-1: Tralokinumab 300 mg + TCS/TCI Q2W (maintained in 48.1% of patients) EASI-75: Tralokinumab 300 mg Q2W + TCS/TCI (maintained in 82.5% of patients)	[41]
			LEBRIKIZUMAB		
NCT04146363 (ADvocate1)	Phase III	424; Adults and adolescents (12 to <18 years of age, weighing ≥40 kg)	Lebrikizumab 250 mg or placebo, Q2W (monotherapy) for 16 weeks Patients who responded at week 16 were re-randomized to receive lebrikizumab 250 mg Q2W, lebrikizumab 250 mg Q4W or placebo Q2W for 36 additional weeks.	Week 16 results: IGA 0-1 (≥2-point reduction): Lebrikizumab 250 mg Q2W (43.1%), Placebo (12.7%) EASI-75: Lebrikizumab 250 mg Q2W (58.8%), Placebo (16.2%) Week 52 results (ADvocate1 and ADvocate2 pooled): IGA 0-1 (≥2-point reduction): Lebrikizumab 250 mg Q2W (71.2%), Lebrikizumab 250 mg Q4W (76.9%), Placebo (47.9%) EASI-75: Lebrikizumab 250 mg Q2W (78.4%), Lebrikizumab Q4W (81.7%), Placebo (66.4%)	[42] [43]
NCT04178967 (ADvocate2)	Phase III	427; Adults and adolescents (12 to <18 years of age, weighing ≥40 kg)	Lebrikizumab 250 mg or placebo, Q2W (monotherapy) for 16 weeks Patients who responded at week 16 were re-randomized to receive lebrikizumab 250 mg Q2W, lebrikizumab 250 mg Q4W or placebo Q2W for 36 additional weeks.	Week 16 results: IGA 0-1 (≥2-point reduction): Lebrikizumab 250 mg Q2W (33.2%), Placebo (10.8%) EASI-75: Lebrikizumab 250 mg Q2W (52.1%), Placebo (18.1%) Week 52 results (ADvocate1 and ADvocate2 pooled): IGA 0-1 (≥2-point reduction): Lebrikizumab 250 mg Q2W (71.2%), Lebrikizumab 250 mg Q4W (76.9%), Placebo (47.9%) EASI-75: Lebrikizumab 250 mg Q2W (78.4%), Lebrikizumab Q4W (81.7%), Placebo (66.4%)	[42] [43]
NCT04250337 (ADhere)	Phase III	211 Adults; adolescents (aged ≥12 to <18 years weighing ≥40 kg)	Lebrikizumab 250 mg Q2W or placebo Q2W in combination with TCS for 16 weeks.	Week 16 results: IGA 0-1: Lebrikizumab 250 mg + TCS, Q2W (41.2%), Placebo + TCS (22.1%) EASI-75: Lebrikizumab 250 mg Q2W (69.5%), Placebo (42.2%)	[44]
NCT04392154 (ADjoin)	Phase III	267; Patients who achieved IGA 0,1 or EASI-75 at 16 weeks in ADvocate 1/2 and ADhere enrolled in ADjoin	Lebrikizumab 250 mg Q2W or Q4W + TCS	Week 40 results: IGA 0-1: Lebrikizumab 250 mg + TCS, Q2W/Q4W maintained an IGA 0/1 response in 75.4% and 86.8%, respectively EASI-75: Lebrikizumab 250 mg + TCS Q2W/Q4W maintained EASI-75 response in 85.6% and 81.2%, respectively Week 104 results: ADvocate1/2 to ADjoin: IGA 0-1: Lebrikizumab 250 mg + TCS, Q2W/Q4W maintained an IGA 0/1 response in 86% and 76%, respectively EASI-75: Lebrikizumab 250 mg + TCS Q2W/Q4W maintained EASI-75 response in 96% in both groups ADhere to ADjoin: IGA 0-1: Lebrikizumab 250 mg + TCS, Q2W/Q4W maintained an IGA 0/1 response in 84% and 79%, respectively EASI-75: Lebrikizumab 250 mg + TCS Q2W/Q4W maintained EASI-75 response in 95% and 96%, respectively	[45] [46]
NCT04626297 (ADopt-VA)	Phase III	188; Adults	Lebrikizumab 250 mg Q2W	Lebrikizumab did not negatively impact immune responses for Tdap or MCV vaccines.	[47]
NCT04250350 (ADore)	Phase III	206; Adolescents (≥12 to <18 years old, weighing ≥ 40 kg)	Lebrikizumab 250 mg Q2W through week 52 TCS, TCI, topical PDE-4 inhibitor allowed as rescue treatment.	Week 16 results: IGA 0-1: Lebrikizumab 250 mg Q2W (46.3%) EASI-75: Lebrikizumab 250 mg Q2W (73.2%) Week 52 results: IGA 0-1: Lebrikizumab 250 mg Q2W (62.6%) EASI-75: Lebrikizumab 250 mg Q2W (81.9%)	[48]
**Anti-IL31RA antibodies**					
			NEMOLIZUMAB		
JapicCTI-183894 (Study-JP02)	Phase III	88; Adults and adolescents (aged ≥ 13 years, weighing ≥ 30.0 kg)	Nemolizumab 60 mg Q4W Patients completing 16 weeks could enter a 52-week extension period; no additional selection criteria imposed. All patients received nemolizumab 60 mg Q4W up to week 64.	Week 16 results: sIGA 0-1 (≥2-point reduction): Nemolizumab Q4W (8.0%) EASI-75: Nemolizumab Q4W (33%) Week 52 results: sIGA 0-1 (≥2-point reduction): Nemolizumab Q4W (12.5%) EASI-75: Nemolizumab Q4W (52.3%)	[49]
JapicCTI-173740 (Study-JP01)	Phase III	215; Adults and adolescents (aged ≥ 13 years, weighing ≥ 30.0 kg)	Nemolizumab 60 mg or placebo Q4W. Previous medications for AD (TCS, TCI, oral antihistamines) unchanged; 16 weeks. Patients completing 16 weeks could enter a 52-week extension period; no additional selection criteria imposed. All patients received nemolizumab 60 mg Q4W up to week 64 (nemolizumab/nemolizumab and placebo/nemolizumab groups).	Week 16 results: sIGA 0-1 (≥2-point reduction): Nemolizumab Q4W+ TCS/TCI (5.6%), Placebo + TCS/TCI (5.6%) EASI-75: Nemolizumab Q4W + TCS/TCI (25.9%), Placebo + TCS/TCI (18.1%) Week 68 results: sIGA 0-1 (≥2-point reduction): Nemolizumab/Nemolizumab + TCS/TCI (28.7%), Placebo/Nemolizumab + TCS/TCI (16.7%) EASI-75: Nemolizumab/Nemolizumab + TCS/TCI (66.4%), Placebo/Nemolizumab + TCS/TCI (59.7%) The least-squares mean percent change from baseline in the pruritus VAS score was −42.8% in the group treated with nemolizumab and −21.4% in the placebo group; the least-squares mean percent change at EASI was −45.9% in the nemolizumab group and −33.2% in the placebo group.	[50] [51] [49]
jRCT2080225289	Phase III	89; Children aged ≥6 and <13 years	Nemolizumab 30 mg Q4W or placebo Q4W; TCS allowed	Week 16 results: sIGA 0-1 (≥2-point reduction): Nemolizumab (17.8%), Placebo (9.1%) EASI-75: Nemolizumab Q4W (31.1%), Placebo (20.5%)	[52]
NCT03985943 (ARCADIA 1)	Phase III	941; Adults and adolescents (aged ≥ 12 years)	Nemolizumab 30 mg Q4W + TCS/TCI or placebo Q4W + TCS/TCI	Week 16 results: IGA 0-1: Nemolizumab Q4W+ TCS/TCI (35.6%), Placebo + TCS/TCI (24.6%) EASI-75: Nemolizumab Q4W + TCS/TCI (43.5%), Placebo + TCS/TCI (29.0%)	[53]
NCT03989349 (ARCADIA 2)	Phase III	787; Adults and adolescents (aged ≥ 12 years)	Nemolizumab 30 mg Q4W +TCS/TCI or placebo Q4W + TCS/TCI	Week 16 results: IGA 0-1: Nemolizumab Q4W+ TCS/TCI (37.7%), Placebo + TCS/TCI (26.0%) EASI-75: Nemolizumab Q4W + TCS/TCI (42.1%), Placebo + TCS/TCI (30.2%)	[53]
**Anti-OX40 antibodies**					
ROCATINLIMAB					
NCT05899816 NCT05398445 NCT05651711 NCT05882877 NCT05704738 NCT05724199 NCT05633355	Phase III	Trials in progress	Diverse treatment regimens	Phase III trial results have not yet been published. Results from the Phase II trial are detailed in the text.	
AMLITELIMAB					
NCT06130566 NCT06181435	Phase III	Trials in progress	Diverse treatment regimens	Phase III trial results have not yet been published. Results from the Phase II trial are detailed in the text.	

Abbreviations: EASI, Eczema Area and Severity Index; HECSI, Hand Eczema Severity Index, IGA, Investigator’s Global Assessment; SCORAD, Scoring Atopic Dermatitis; sIGA, static Investigator’s Global Assessment; SIS, systemic immunosuppressant; TCI, topical calcineurin inhibitors; TCS, topical corticosteroids; LSMD, Least Squares Mean Difference; MCV, Meningococcal (Groups A, C, Y, and W-135) vaccine; PDE-4 inhibitor, phosphodiesterase-4 inhibitors; SIS, systemic immunosuppressant; Tdap, Diphtheria/Tetanus Toxoids/Pertussis vaccine; QW, every week; Q2W, every two weeks; Q4W, every 4 weeks; Q8W, every eight weeks, VAS, visual analogue scale.

## Data Availability

As reported in Section 2.

## Supplementary Materials

The following supporting information can be downloaded at: https://www.mdpi.com/article/10.3390/jcm13144001/s1, Table S1: Summary of phase III and IV clinical trials on biologic drugs in AD treatment (ClinicalTrials.gov; JapicCTI/jRCT as of 11 January 2024).
