# Supplementary material for: A Comprehensive Review of Biologics in Phase III and IV Clinical Trials for Atopic Dermatitis
Source: J Clin Med. 2024 Jul 9;13(14):4001. doi: 10.3390/jcm13144001 (PMC11277805; doi:10.3390/jcm13144001)
Supplement: Supplementary file 1 [file jcm-13-04001-s001.zip › jcm-3073326-supplementary.pdf]

Supplementary Table S1. Summary of phase III and IV clinical trials on biologic drugs in AD treatment (Clinical Trials.gov; JapicCTI as of 11 January 2024).

Study No.	Study Title	Drug	Status	Clinical Trial phase
NCT03054428	Efficacy and Safety of Dupilumab in Participants ≥12 to <18 Years of Age, With Moderate-to-severe Atopic Dermatitis	Dupilumab	COMPLETED	III
NCT04417894	A Study to Evaluate the Efficacy and Safety of Dupilumab in Adult and Adolescent Patients With Moderate-to-Severe Atopic Hand and Foot Dermatitis (Liberty-AD-HAFT)	Dupilumab	COMPLETED	III
NCT05590585	Dupilumab in Adolescent and Adult Skin of Color Participants: Open-label Moderate-to-severe Eczema Trial	Dupilumab	RECRUITING	IV
NCT05042258	Using Dupilumab to Improve Circadian Function, Sleep and Pruritus in Children With Moderate/Severe Atopic Dermatitis	Dupilumab	NOT YET RECRUITING	IV
NCT04520308	An Open-label, Single-arm Longitudinal Study With Dupilumab for Patients With Atopic Dermatitis	Dupilumab	UNKNOWN STATUS	IV
NCT04358224	The Utility of Functionally Relevant Signature Genes in Assessing the Clinical Outcomes of Dupilumab Treatment	Dupilumab	COMPLETED	IV
NCT03389893	Effect of Dupilumab (Anti-IL4Rα) on the Host-Microbe Interface in Atopic Dermatitis	Dupilumab	TERMINATED	IV
NCT05265234	Dupilumab for Atopic Dermatitis Monitored With Noninvasive Imaging.	Dupilumab	RECRUITING	IV
NCT05858619	Molecular Signatures of Cutaneous Dupilumab Response	Dupilumab	RECRUITING	IV
NCT05268107	Ethnic Differences in Mechanisms of Action of Dupilumab	Dupilumab	RECRUITING	IV
NCT03293030	Immunogenetic Profiling of Dupilumab for the Treatment of Atopic Dermatitis	Dupilumab	RECRUITING	IV
NCT02755649	A Study to Assess the Efficacy and Safety of Dupilumab in Participants With Severe Atopic Dermatitis (AD) That Are Not Controlled With Oral Cyclosporine A (CSA) or for Those Who Cannot Take Oral CSA Because it is Not Medically advisable	Dupilumab	COMPLETED	III
NCT02277769	Study of Dupilumab (REGN668/SAR231893) Monotherapy Administered to Adult Patients With Moderate-to-Severe Atopic Dermatitis	Dupilumab	COMPLETED	III

NCT02277743	Study of Dupilumab Monotherapy Administered to Adult Patients With Moderate-to-Severe Atopic Dermatitis	Dupilumab	COMPLETED	III
NCT04033367	SAR231893-LPS15497- "Dupilumab Effect on Sleep in AD Patients"	Dupilumab	COMPLETED	IV
NCT02260986	Study to Assess the Efficacy and Long-term Safety of Dupilumab (REGN668/SAR231893) in Adult Participants With Moderate-to-Severe Atopic Dermatitis	Dupilumab	COMPLETED	III
NCT02395133	A Study to Confirm the Efficacy and Safety of Different Dupilumab Dose Regimens in Adults With Atopic Dermatitis (AD)	Dupilumab	COMPLETED	III
NCT03667014	The Impact of Dupilumab on Quality of Life in Moderate to Severe Atopic Dermatitis Patients	Dupilumab	COMPLETED	IV
NCT04718870	Dupilumab-PEdiatric Skin Barrier Function and LIpidomics STudy in Patients With Atopic Dermatitis	Dupilumab	COMPLETED	IV
NCT02612454	Study to Assess the Long-term Safety of Dupilumab Administered in Participants ≥6 Months to <18 Years of Age With Atopic Dermatitis (AD)	Dupilumab	ACTIVE, NOT RECRUITING (with partial results)	III
NCT06004986	DUPIlumab Dose REDUCtion in Patients With Controlled Atopic Eczema	Dupilumab	RECRUITING	IV
NCT06116526	Dupilumab De-escalation in Pediatric Atopic Dermatitis	Dupilumab	NOT YET RECRUITING	IV
NCT04447417	Dupilumab Skin Barrier Function Study in Atopic Dermatitis	Dupilumab	COMPLETED	IV
NCT05285839	Dupixent and Narrowband UVB for Atopic Dermatitis	Dupilumab	NOT YET RECRUITING	IV
NCT05624112	Dupilumab Skin Barrier Function and Lipidomics Study in Atopic Dermatitis in China	Dupilumab	RECRUITING	IV
NCT05680298	Dupilumab-pediatric Skin Barrier Function and Lipidomics Study in Patients With Atopic Dermatitis in China	Dupilumab	ACTIVE, NOT RECRUITING	IV
NCT04678882	Dupilumab in Japanese Patients With Atopic Dermatitis	Dupilumab	ACTIVE, NOT RECRUITING	III
NCT03912259	Evaluation of Dupilumab in Chinese Adult Patients With Moderate to Severe Atopic Dermatitis	Dupilumab	COMPLETED	III
NCT04823130	Dupilumab Effect on Pruritus Neuro-mechanisms in Patients With Atopic Dermatitis	Dupilumab	COMPLETED	IV

NCT05983068	A Study of Long-term Effect of Dupilumab on Skin Barrier Function in Pediatric Participants With Atopic Dermatitis	Dupilumab	NOT YET RECRUITING	IV
NCT03346434	Safety, Pharmacokinetics and Efficacy of Dupilumab in Patients ≥6 Months to <6 Years With Moderate-to-Severe Atopic Dermatitis (Liberty AD PRESCHOOL)	Dupilumab	COMPLETED	III
NCT05642208	Dupilumab Step-down Strategy to Maintain Remission in Adult and Adolescents Patients With Atopic Dermatitis	Dupilumab	RECRUITING	IV
NCT01949311	Open-label Study of Dupilumab in Patients With Atopic Dermatitis	Dupilumab	COMPLETED	III
NCT03345914	Study to Investigate the Efficacy and Safety of Dupilumab Administered With Topical Corticosteroids (TCS) in Participants ≥6 to <12 Years With Severe Atopic Dermatitis (AD)	Dupilumab	COMPLETED	III
NCT06026891	Study of MG-K10 Humanized Monoclonal Antibody Injection in Patients With Atopic Dermatitis	MG-K10	ACTIVE, NOT RECRUITING	III
NCT05265923	A Study of CM310 in Patients With Moderate-to-Severe Atopic Dermatitis	CM310	RECRUITING	III
NCT06173284	Evaluation of 611(Recombinant Humanized Anti-interleukin-4 Receptor Alpha IgG4 Monoclonal Antibody) in Chinese Adults With Moderate to Severe Atopic Dermatitis	611	RECRUITING	III
NCT05958407	A 32-week Trial to Evaluate the Efficacy and Safety of Tralokinumab in Subjects With Moderate-to-severe Atopic Hand Eczema Who Are Candidates for Systemic Therapy	Tralokinumab	RECRUITING	III
NCT05378698	Effects of Tralokinumab in the Skin: an Immunologic and Molecular Investigation	Tralokinumab	NOT YET RECRUITING	III
NCT05194540	Tralokinumab Administered With Device A in Adults and Adolescents With Moderate-to-severe Atopic Dermatitis	Tralokinumab	COMPLETED	III
NCT04587453	Tralokinumab in Combination With Topical Corticosteroids in Japanese Subjects With Moderate-to-severe Atopic Dermatitis	Tralokinumab	COMPLETED	III
NCT03160885	Tralokinumab Monotherapy for Moderate to Severe Atopic Dermatitis - ECZTRA 2 (ECZema TRAlokinumab Trial no. 2)	Tralokinumab	COMPLETED	III
NCT03131648	Tralokinumab Monotherapy for Moderate to Severe Atopic Dermatitis - ECZTRA 1 (ECZema TRAlokinumab Trial no. 1)	Tralokinumab	COMPLETED	III

NCT03526861	Tralokinumab Monotherapy for Adolescent Subjects With Moderate to Severe Atopic Dermatitis - ECZTRA 6 (ECZema TRAlokinumab Trial no. 6).	Tralokinumab	COMPLETED	III
NCT03761537	Tralokinumab in Combination With Topical Corticosteroids in Subjects With Severe Atopic Dermatitis - ECZTRA 7	Tralokinumab	COMPLETED	III
NCT03587805	Long-term Extension Trial in Subjects With Atopic Dermatitis Who Participated in Previous Tralokinumab Trials - ECZTEND	Tralokinumab	ACTIVE, NOT RECRUITING (with partial results)	III
NCT03363854	Tralokinumab in Combination With Topical Corticosteroids for Moderate to Severe Atopic Dermatitis - ECZTRA 3	Tralokinumab	COMPLETED	III
NCT05149313	A Study of Lebrikizumab in Combination With Topical Corticosteroids in Participants Having Atopic Dermatitis (AD) That Are Not Adequately Controlled or Non-eligible for Cyclosporine	Lebrikizumab	ACTIVE, NOT RECRUITING	III
NCT05735483	A Study to Assess the Long-Term Safety and Efficacy of Lebrikizumab (LY3650150) in Participants 6 Months to <18 Years of Age With Moderate-to-Severe Atopic Dermatitis	Lebrikizumab	RECRUITING	III
NCT04760314	A Study of Lebrikizumab (LY3650150) in Combination With Topical Corticosteroids in Japanese Participants With Moderate-to-Severe Atopic Dermatitis	Lebrikizumab	COMPLETED	III
NCT05559359	A Study of Lebrikizumab (LY3650150) in Participants 6 Months to <18 Years of Age With Moderate-to-Severe Atopic Dermatitis	Lebrikizumab	RECRUITING (with partial results)	III
NCT05990725	Effectiveness and Safety of Lebrikizumab Treatment in Adults and Adolescents With Moderate-to-Severe Atopic Dermatitis	Lebrikizumab	NOT YET RECRUITING	III
NCT05916365	Long-term Safety and Efficacy of Lebrikizumab in Adult and Adolescent Participant With Moderate-to-Severe Atopic Dermatitis	Lebrikizumab	RECRUITING	III
NCT04392154	Long-term Safety and Efficacy Study of Lebrikizumab (LY3650150) in Participants With Moderate-to-Severe Atopic Dermatitis (ADjoin)	Lebrikizumab	ACTIVE, NOT RECRUITING (with partial results)	III
NCT04178967	Evaluation of the Efficacy and Safety of Lebrikizumab (LY3650150) in Moderate to Severe Atopic Dermatitis	Lebrikizumab	COMPLETED	III
NCT04146363	Evaluation of the Efficacy and Safety of Lebrikizumab (LY3650150) in Moderate to Severe Atopic Dermatitis (ADvocate1)	Lebrikizumab	COMPLETED	III

NCT04250350	Study to Assess the Safety and Efficacy of Lebrikizumab (LY3650150) in Adolescent Participants With Moderate-to-Severe Atopic Dermatitis	Lebrikizumab	COMPLETED	III
NCT04250337	Safety and Efficacy of Lebrikizumab (LY3650150) in Combination With Topical Corticosteroid in Moderate-to-Severe Atopic Dermatitis.	Lebrikizumab	COMPLETED	III
NCT05369403	A Study of Lebrikizumab (LY3650150) in Adult and Adolescent Participants With Moderate-to-Severe Atopic Dermatitis Previously Treated With Dupilumab	Lebrikizumab	RECRUITING	III
NCT05372419	A Study of (LY3650150) Lebrikizumab to Assess the Safety and Efficacy of Adult and Adolescent Participants With Moderate-to-Severe Atopic Dermatitis and Skin of Color	Lebrikizumab	RECRUITING	III
NCT04626297	A Study of Lebrikizumab (LY3650150) on Vaccine Response in Adults With Atopic Dermatitis (ADopt-VA)	Lebrikizumab	COMPLETED	III
NCT03989349	Efficacy & Safety of Nemolizumab in Subjects With Moderate-to-Severe Atopic Dermatitis	Nemolizumab	COMPLETED	III
NCT03985943	Efficacy and Safety of Nemolizumab in Subjects With Moderate-to-Severe Atopic Dermatitis	Nemolizumab	COMPLETED	III
NCT03989206	Long-term Safety and Efficacy of Nemolizumab With Moderate-to-severe Atopic Dermatitis	Nemolizumab	ACTIVE, NOT RECRUITING	III
JapicCTI-183894	Nemolizumab plus topical agents in patients with atopic dermatitis (AD) and moderate-to-severe pruritus: 68 weeks study*	Nemolizumab	COMPLETED	III
JapicCTI-173740	Nemolizumab plus topical agents in patients with atopic dermatitis (AD) and moderate-to-severe pruritus: 68 weeks study*	Nemolizumab	COMPLETED	III
jRCT2080225289	Efficacy and safety of nemolizumab in paediatric patients aged 6-12 years with atopic dermatitis with moderate-to-severe pruritus*	Nemolizumab	COMPLETED	III
NCT05899816	A Study Assessing Rocatinlimab on Vaccine Antibody Response in Moderate-to-severe Atopic Dermatitis (AD) (ROCKET - VOYAGER)	Rocatinlimab	RECRUITING	III
NCT05398445	A Study Evaluating Rocatinlimab in Moderate-to-severe Atopic Dermatitis (ROCKET-IGNITE)	Rocatinlimab	RECRUITING	III
NCT05651711	A Study Assessing Rocatinlimab (AMG 451) Monotherapy in Moderate-to-severe Atopic Dermatitis (AD) (ROCKET-Horizon)	Rocatinlimab	RECRUITING	III

NCT05882877	A Study to Assess Long-term Safety, Tolerability, and Efficacy of Rocatinlimab in Adult and Adolescent Participants With Moderate-to-severe Atopic Dermatitis (AD)	Rocatinlimab	RECRUITING	III
NCT05704738	A Study to Evaluate Rocatinlimab (AMG 451) in Adolescent Subjects With Moderate-to-severe Atopic Dermatitis (AD)	Rocatinlimab	RECRUITING	III
NCT05633355	A Study to Assess the Safety, Tolerability, and Efficacy of Rocatinlimab in Adolescent Participants With Moderate-to-severe Atopic Dermatitis (AD)	Rocatinlimab	RECRUITING	III
NCT05724199	A Study Assessing Rocatinlimab in Combination With Topical Corticosteroid and/or Topical Calcineurin Inhibitors in Adult Participants With Moderate-to-severe Atopic Dermatitis (AD)	Rocatinlimab	RECRUITING	III
NCT06130566	A Study to Evaluate the Efficacy and Safety of Subcutaneous Amlitelimab Monotherapy Compared With Placebo in Participants Aged 12 Years and Older With Moderate-to-severe Atopic Dermatitis	Amlitelimab	RECRUITING	III
NCT06181435	A Study to Evaluate the Efficacy and Safety of Subcutaneous Amlitelimab Monotherapy Compared With Placebo in Participants Aged 12 Years and Older With Moderate-to-severe Atopic Dermatitis (COAST 2)	Amlitelimab	RECRUITING	III

* based on article title [51], exact study title not found

References

51. Kabashima, K.; Matsumura, T.; Hayakawa, Y.; Kawashima, M. Clinically meaningful improvements in cutaneous lesions and quality of life measures in patients with atopic dermatitis with greater pruritus reductions after treatment with 60 mg nemolizumab subcutaneously every 4 weeks: Subgroup analysis from a phase 3, randomized, controlled trial. *J. Dermatol. Treat.* **2023**, *34*, 2177096.
